# Nd^3+^-doped transparent tellurite ceramics bulk lasers

**DOI:** 10.1038/s41598-018-22922-5

**Published:** 2018-03-15

**Authors:** Morgane Dolhen, Masayuki Tanaka, Vincent Couderc, Sébastien Chenu, Gaëlle Delaizir, Tomokatsu Hayakawa, Julie Cornette, François Brisset, Maggy Colas, Philippe Thomas, Jean-René Duclère

**Affiliations:** 1 Univ. Limoges, CNRS, IRCER, UMR 7315, F-87000 Limoges, France; 2 Field of Advanced Ceramics, Department of Life Science and Applied Chemistry, Nagoya Institute of Technology, Gokiso, Showa, Nagoya, 466-8555 Japan; 3 Institut XLIM, UMR 7252 CNRS – Université de Limoges, 123, Avenue Albert Thomas, 87060 Limoges Cedex, France; 4 Institut de Chimie Moléculaire et des Matériaux d’Orsay (ICMMO), UMR 8182 CNRS Orsay, France

## Abstract

We report on the laser emission of the polycrystalline ceramic obtained from the full and congruent crystallization of the parent glass 1Nd^3+^:75TeO_2_-12.5Bi_2_O_3_-12.5Nb_2_O_5_ composition. In particular, the current work underlines the importance of carefully controlling the heat treatment in order to solely crystallize the Bi_0.8_Nb_0.8_Te_2.4_O_8_ cubic phase and consequently avoid the formation of the BiNbTe_2_O_8_ orthorhombic phase that would be detrimental for optical purpose. The structure, microstructure and photoluminescence properties of the resulting transparent tellurite ceramics are characterized. The continuous-wave and gain-switching laser performances reveal that the emission remains perfectly single transversal mode in the range of pump powers explored. The maximum output power achieved was ~28.5 mW, for a pump power threshold of ~67 mW, and with associated efficiency and slope efficiency of ~22.5% and ~50%, respectively. These data definitely stand among the best results obtained so far for bulk laser tellurite materials and thus demonstrate the potential of such polycrystalline transparent ceramics as optically active materials. Finally, the laser emission characteristics in pulsed regime, at low and high repetition rates, are also provided: more than 6.5 W of peak power at a repetition rate of 728 kHz can be obtained.

## Introduction

Tellurite based-glasses present numerous advantages, such as a large transparency window up to ~6 µm, a rather low melting point and excellent third-order non-linear optical properties^1^. Also, tellurite materials are very attractive for their high rare earth ions solubility^2^, coupled with low phonon energies (~600–700 cm^−1^)^1–4^. These are key parameters which will favour radiative transitions, further permitting laser emission.

So far, all the literature dedicated to tellurite bulk lasers is still rather scarce, as it was recently reviewed in^5^, and most of all, is limited to bulk glass lasers^6–21^. The most convenient way to sort all these contributions remains to classify them into three categories, which find their origin from the nature of the rare earth ions employed. Hence, papers dealing with Nd^3+^-doped tellurite glass bulk lasers are the most abundant^6–15^: except one of them which treats the lasing properties at 1.37 μm, they are all related to laser emission around 1064 nm. Then, one can find articles reporting on Ho^3+^-doped, Tm^3+^-doped and also Tm^3+^-Ho^3+^ co-doped tellurite glasses for laser emission around 2 μm^16–19^. Finally, the last category regroups papers focused on Yb^3+^-doped tellurite glasses, emitting in the 0.9–1.1 μm range, like in^20^.

Besides that, there is some important and recent research activity concentrated on the study of the optical properties of tellurite glass-ceramics, considered as promising materials for lasers, especially in the near and mid-infrared^21–23^. However, to the authors knowledge, laser emission in bulk tellurite glass-ceramics has not yet been reported.

On another hand, a very interesting and innovative way of producing some transparent ceramics is the full congruent crystallization method, starting from the parent glass^24,25^. In particular, such route was recently adapted to tellurite materials, and more specifically to the 75TeO_2_-12.5Nb_2_O_5_-12.5Bi_2_O_3_ composition^26^.

Thus, up to date, there are no reports dealing with the laser characteristics of such transparent tellurite ceramics obtained by full congruent crystallization from the parent glass, and the current work aims at evaluating this. In the first part of the paper, the elaborated bulk tellurite ceramics are characterized (structural and microstructural data, as well as optical transparency and photoluminescence properties, are presented and discussed). In the second part, the lasing emission, both in continuous wave (cw) and pulsed regimes, is reported. Details about the spatial shape of the laser beam, the spectral characteristics of the emission, the effect of the output couplers employed and the corresponding impact on the measured lasing efficiency, are provided.

## Methods

**Figure 1:**
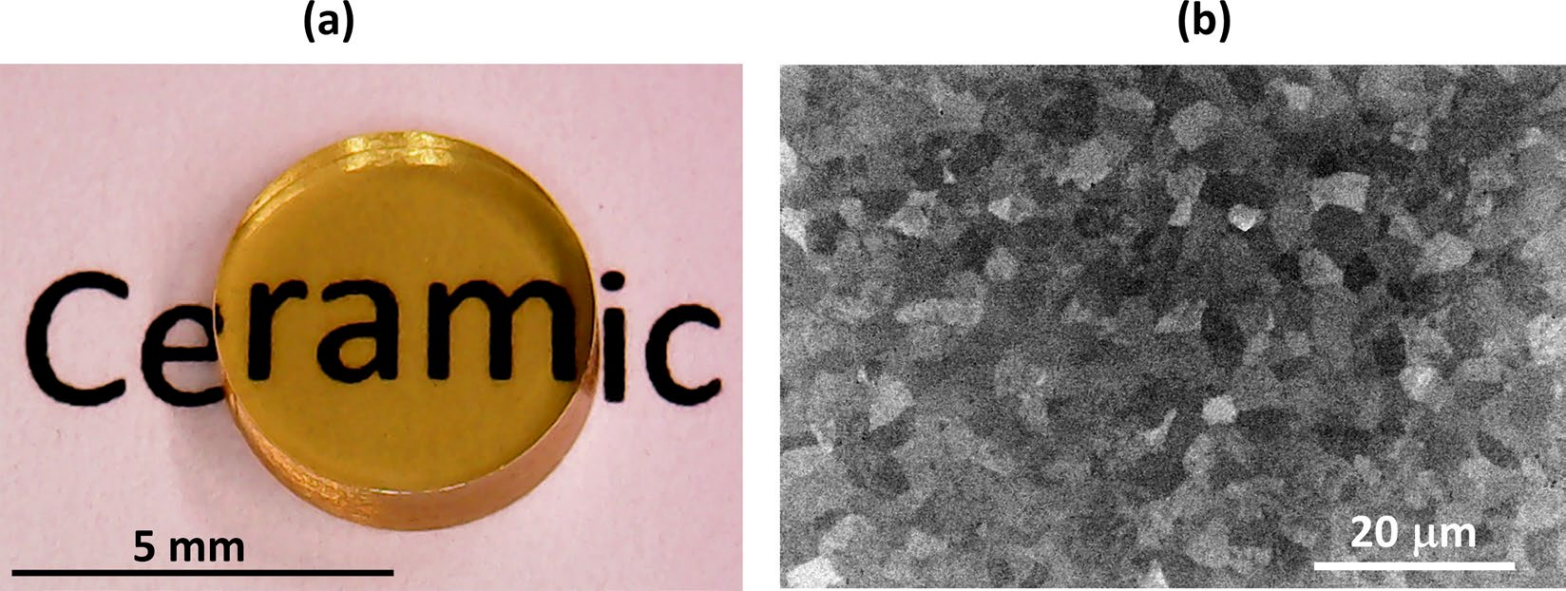
(**a**) Picture taken of a typical bulk tellurite ceramic, placed above a text, and dedicated for performing laser emission tests. (**b**) SEM observation of the polished surface, revealing the ceramic grains (please note that the contrast of the picture was enhanced and that no chemical etching was used).

Glasses were prepared by melting intimately specific quantities of TeO_2_, Nb_2_O_5_, Bi_2_O_3_ and Nd_2_O_3_ dried powders, in platinum crucibles, at 850 °C. TeO_2_ (Alfa-Aesar, 99.99%), Nb_2_O_5_ (Sigma-Aldrich, 99.99%), Bi_2_O_3_ (Sigma-Aldrich, 99.9%) and Nd_2_O_3_ (Sigma-Aldrich, 99.999%) are all commercial products. The chemical composition tested in this work is: 75TeO_2_-12.5Nb_2_O_5_-12.5Bi_2_O_3_, containing a 1 mol. % in Nd^3+^ as an added “dopant”. The powder mixture was melted during 1 h, with four stirring sessions, in order to reach a sufficiently good homogeneity of the final glasses. The melts were air-quenched into a specific brass ring, deposited over a preheated brass block, to obtain cylindrical samples (5 mm in diameter and ~4 mm thick after polishing). A typical picture of these samples, after the sawing and polishing steps, is given in Fig. 1(a).

Before sawing and polishing, the samples were all annealed 20 °C below their glass transition temperature (T_g_) for 3 h in order to strongly release the stresses resulting from the thermal quenching (slow heating and cooling rates (2 °C/min) were also applied). X-ray diffraction was finally used to verify the glassy state of the samples.

The full ceramization process consisted in a simple heat-treatment, during 5 or 15 min at 510 °C, under atmospheric conditions, using both heating and cooling rates fixed at 10°/min.

The surface microstructure of the polished samples was observed using a Quanta 450 FEG Scanning Electron Microscope (SEM), whereas the Electron Back-Scattered Diffraction (EBSD) map was recorded using an OIM TSL/EDAX system mounted on a FEG-SEM (Zeiss SUPRA 55 VP).

X-ray diffraction data were collected using a D8 Bruker diffractometer, operated in the θ–2θ mode (2θ range 25°–38°) and equipped with the CuKα radiation. Experiments were conducted as a function of time, at a temperature T = 510 °C (temperature based on our previous work^26^).

Raman spectroscopy measurements were conducted in back-scattering geometry, with an excitation wavelength at 514.532 nm, using a T64000 Jobin-Yvon spectrophotometer operated in triple substractive configuration (1800 grooves/mm) and associated to a liquid nitrogen-cooled CCD detector. Measurements were performed at low power (~30 mW) in order to avoid any damage of the samples. The scattered light was collected through a microscope objective (×50 Low Working Distance), with a minimum spot size of about 1.1 micron in diameter.

Optical transmission measurements of thinner and polished samples (glass and ceramics), without the presence of any R_max_ or any anti-reflective coatings, were carried out in the UV-visible-Near InfraRed (NIR) range (200–3300 nm), at normal incidence, using a Varian Cary 5000 spectrophotometer operated in dual beam mode.

The photoluminescence (PL) properties were measured at room temperature (RT), using a Horiba-Jobin-Yvon Fluorolog 3 spectrofluorimeter. For the recorded steady state fluorescence emission spectra, the data step was fixed to 1 nm, with a 1 s acquisition time. PL decay curves were measured by setting an initial delay of 50 μs (to completely get rid of any residual excitation pulse stray light) and with time interval steps of 10 μs.

**Figure 2:**
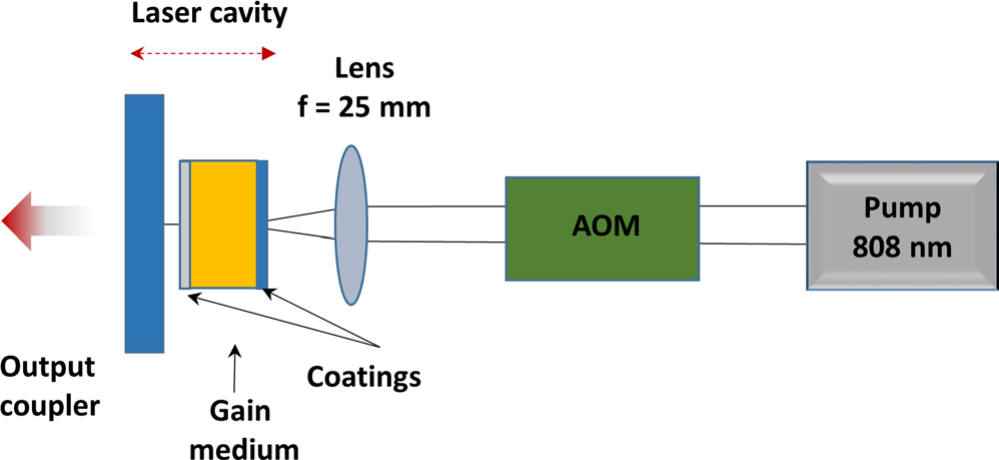
Sketch of the experimental setup employed for the laser emission tests, in the configuration where the acousto-optic modulator (AOM) is placed along the pump beam path.

The experimental setup employed for the lasing tests was already described in^15^, and is also re-sketched on Fig. 2. It is constituted by a single-mode fibered laser diode emitting (cw) radiations at 808 nm which is used as pump beam. An acousto-optic modulator (AOM) is eventually placed on the pump beam pathway to temporally control the energy sent to the gain medium and thus reach an active gain-switched regime. The pump beam is then focused with a beam diameter close to 40 µm in the sample which previously received specific coatings: *i.e*. an anti-reflective coating@808 nm + R_max_@1064 nm on the laser pump diode side and an anti-reflective coating@1064 nm on the laser emission side. The laser cavity is constituted by two plane mirrors surrounding the gain medium, producing an effective cavity length estimated to 12 mm. In that configuration, the cavity stability is obtained thanks to the thermal lens provided by the pump beam. The internal cavity losses are then only introduced by the gain medium diffusion and the anti-reflective coating@1064 nm, and are estimated to be below 1% for one round trip in the cavity, for the 5 min ceramic sample. The output couplers used to extract the laser power from the microcavity are respectively 99.8, 97, 95 and 90% of reflectivity at 1064 nm. Varying the nature of the output coupler served to determine the maximum laser output efficiency.

All data generated or analysed during this study are included in this published article.

## Results and Discussion

### Characterization of the fabricated tellurite ceramics

In our previous work^26^, a large (60 mm in diameter) undoped tellurite glass sample was heat treated at 510 °C for 1 h30, leading to a fully ceramized and optically transparent sample. In the current work, the dwelling time at 510 °C was adapted and drastically shortened (5 or 15 min) mostly due to the strongly reduced size of the sample (Fig. 1(a)), but also because of the insertion of rare-earth ions that slightly decreases the thermal stability of the parent glass.

The microstructure of the sample treated during 5 min was observed by SEM (Fig. 1(b)). One can unambiguously distinguish the grains, proving the full ceramization of the sample, even after such brief heating time. The average size of the grains is roughly 3–5 μm.

**Figure 3:**
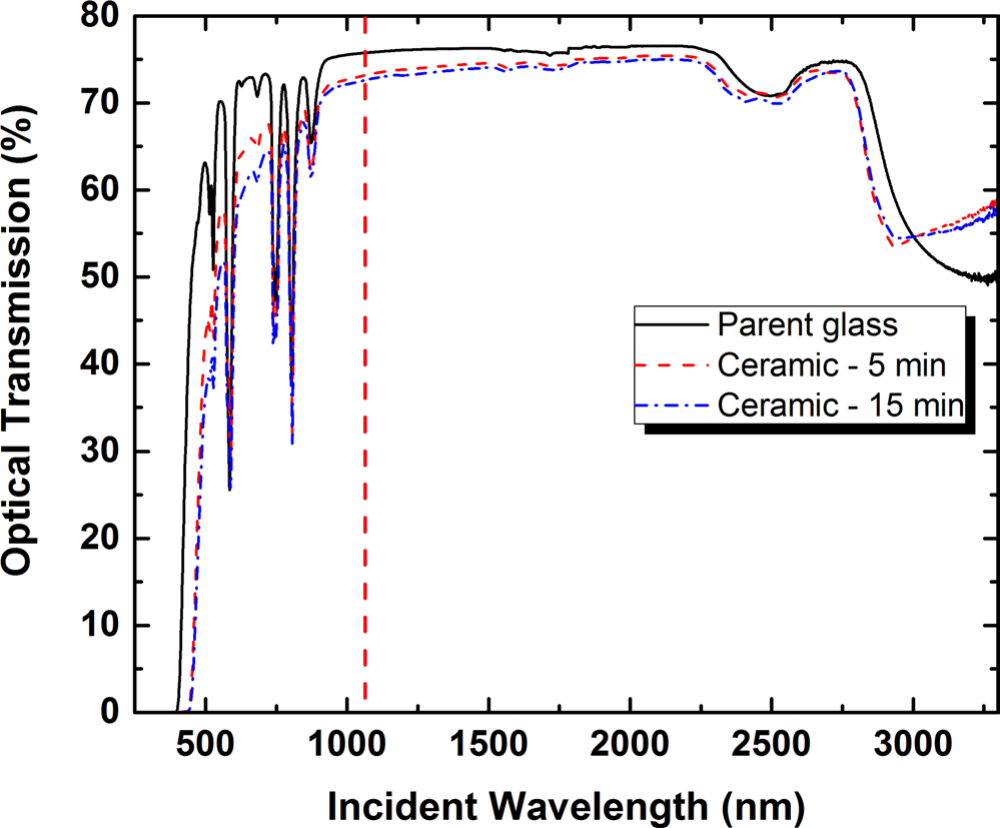
Optical transmission spectra collected for the parent glass, and for the ceramic samples obtained after some specific heat treatments under atmospheric conditions, during 5 min and 15 min at 510 °C, respectively. The vertical dashed line indicates the laser emission wavelength around 1064 nm. All samples display roughly the same thickness after the polishing step (thickness reduced from 4 mm down to 2 mm).

The optical transparency of the fabricated ceramic samples must be then quantified prior to any lasing tests. Figure 3 shows the optical transmission measurements conducted on two polished ceramics (heat treated during 5 and 15 min), in comparison with the transmission data collected for the parent glass. The optical transmission of the two ceramic samples is good, but not as good as the one of the parent Nd^3+^-doped glass. Indeed, light scattering in the visible is clearly present and will tend to fade away rapidly in the NIR: in particular, around 1064 nm (highlighted by the dashed vertical line in Fig. 3), the difference in the optical transmission level is already rather moderate (T = 75.76% for the parent glass, T = 73.17% and T = 72.62%, respectively, for the 5 and 15 min ceramics) and hence, optical performances will appear to be sufficient for observing laser emission in such samples (see further in *Part B*). Beyond 1500 nm, the remaining differences are finally explained by the different refractive indices between the parent glass and the ceramic, as already discussed in^26^. Also, in comparison to the data published in^26^ for undoped tellurite ceramics, the slight deterioration of the optical transparency might be related to the introduction of rare earth ions, but not to the reduction of the heat treatment time. Indeed, when comparing the optical transmission for the samples heat treated during 5 or 15 min, one can evidence some actual degradation of the optical transparency with a longer heat treatment, then clearly indicating the need for carefully controlling the congruent crystallization process (time and temperature must be properly monitored). Thus, all these observations suggest that the “ideal” heat treatment is in fact not yet defined: likely, for such small bulk samples, one will need to heat for even shorter times and/or at slightly lower temperatures in order to reach the optimal transparency of the final doped ceramic samples. Finally, some short comment can be formulated concerning the shape of the OH absorption band around 3000 nm (stretching modes^27^), which clearly differs between the parent glass and the ceramic samples. Indeed, whereas the OH band is broad with a minimum located at λ ~ 3300 nm for the parent glass, the same band sharpens and now offers a minimum located at λ ∼ 2940 nm in the case of ceramic samples. Such behaviour is attributed to the modification of the environment of the OH-groups, concomitant with the ceramization (more symmetric environment in comparison to the glass).

**Figure 4:**
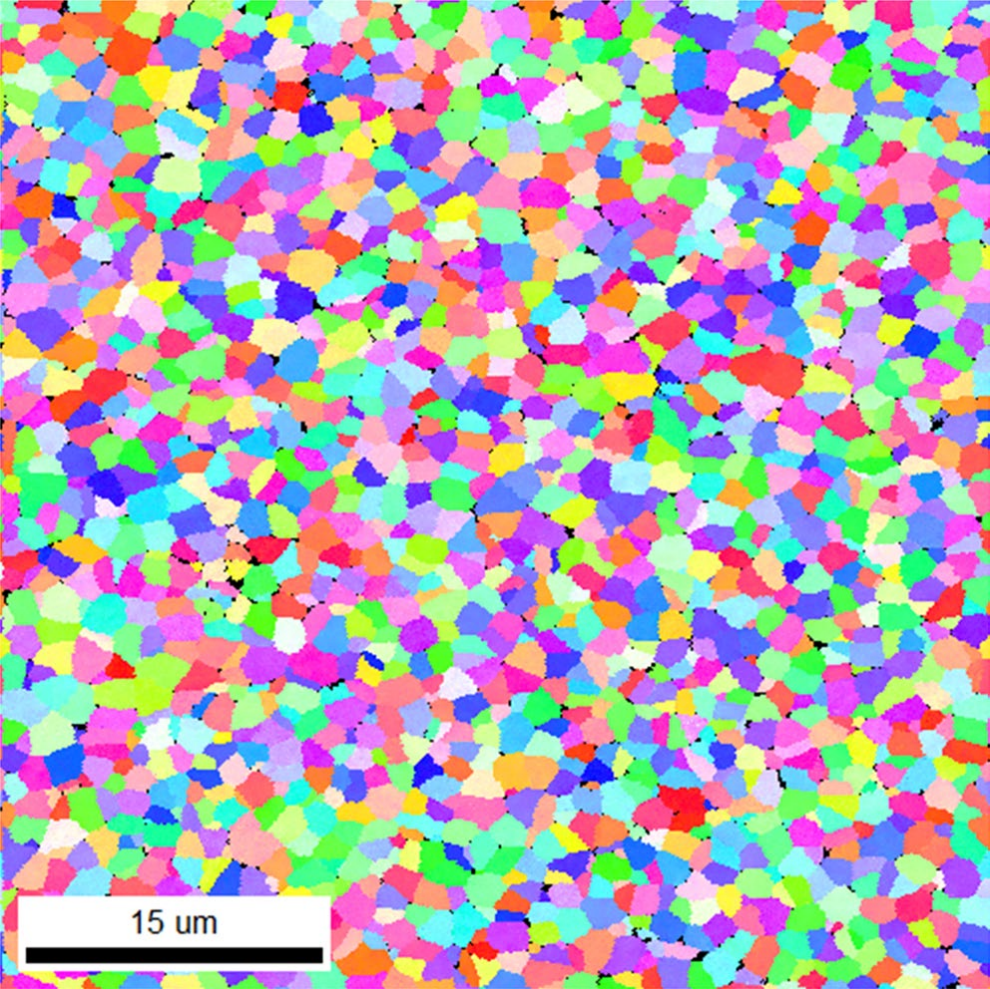
EBSD-SEM map (15 μm scale bar) of the final undoped material, clearly revealing crystalline grains and the associated grain boundaries. Please note the presence of small areas showing a dark contrast and located at the grain boundaries.

Observing the degraded optical transparency of the ceramic samples, mostly in the visible, led us to investigate further their microstructure. As a consequence, an EBSD map was recorded on a similar undoped ceramic sample, elaborated after 5 min spent at 510 °C, under air. As can be seen on Fig. 4, the map consists in slightly smaller grains of ~2 μm (the reduction of size being likely connected to the increase of the thermal stability of the glass, in the case of undoped samples^26^). Most important is the presence of small areas, appearing with a dark contrast and clearly spotted at the grain boundaries. At this stage of our studies, two different plausible explanations could be provided:

- either these dark areas could reflect the presence of some residual glass phase present at the grain boundaries, likely related to the much shorter duration of the heat treatment,
- or these dark areas could reflect the creation of a tiny amount of porosity, concomitant with the transformation of the parent into the final ceramic.
  These two possibilities are now seriously being investigated, as they both could explain the measured optical properties for the produced ceramic samples. However, the second hypothesis appears more credible for three main arguments:
- first, as the parent glass is transformed into the final ceramic, the density respectively varies from 5.97 to 6.24 g/cm^3^, showing thus some noticeable +4.5% increase. For the same occupied volume, denser areas and voids would then be coexisting,
- also, one would expect some improvement of the optical transparency with the longer heat treatment (15 min instead of 5 min), if indeed some residual glass phase was present in the sample elaborated after only 5 min at 510 °C,
- with increasing temperature, the parent glass will transform first into this « anti-glass » structure, before transforming into the final ceramic. Thus, it is much more likely that the remaining phase present at the grain boundaries will correspond to the « anti-glass », rather than to the parent glass. And based on a previous work conducted on another chemical composition within the same TeO_2_-Bi_2_O_3_-Nb_2_O_5_ system^28^, it is known that the « anti-glass » phase should provide some EBSD signature.

**Figure 5:**
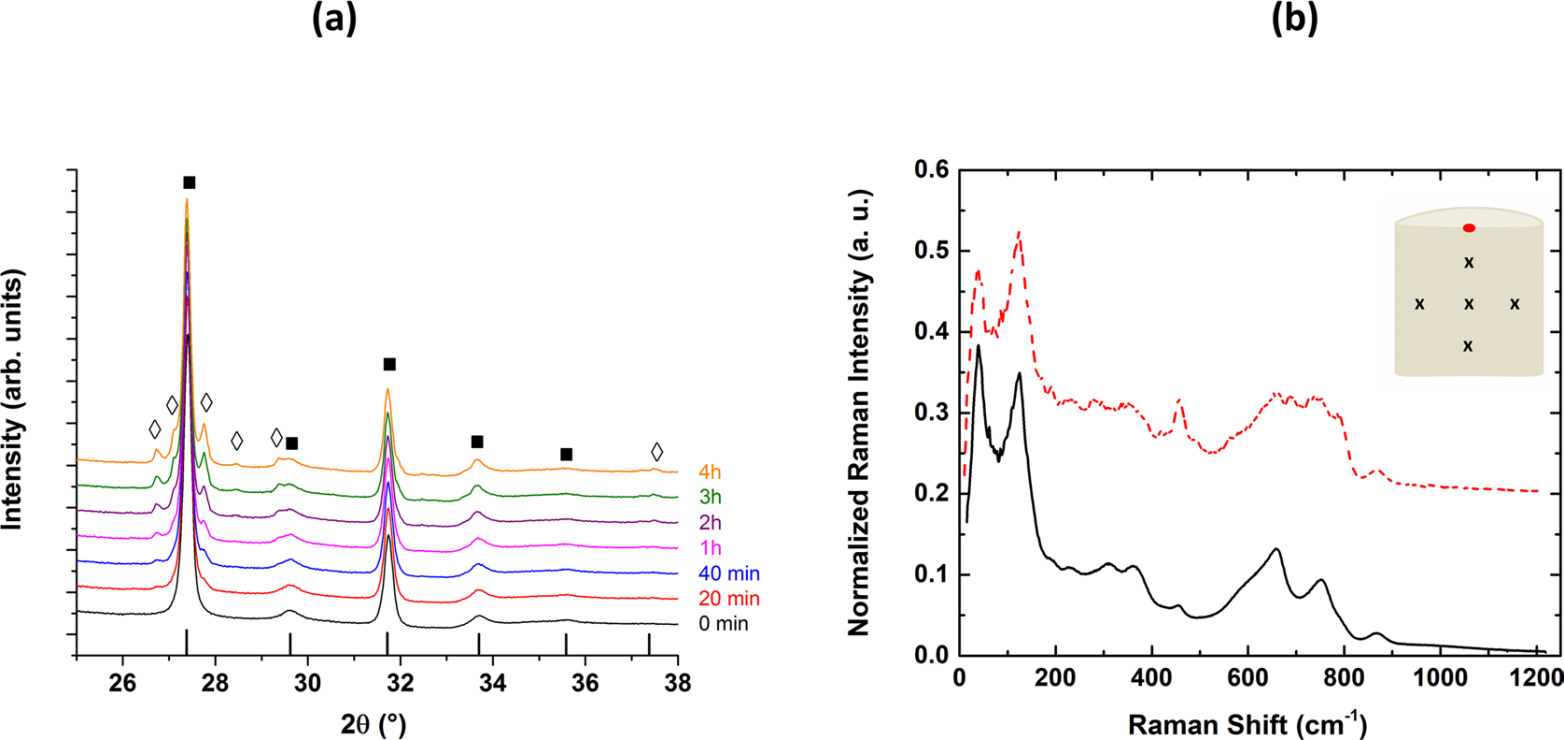
(**a**) θ-2θ XRD data collected as a function of time (from 0 min up to 4 h), at a temperature T = 510 °C, under atmospheric conditions, starting from an initial parent glass under powder form, and displayed in the 25–38° 2θ range. The (■) symbols correspond to the main cubic Bi_0.8_Nb_0.8_Te_2.4_O_8_ crystal phase (I*a*-3 space group), whereas the ◇ symbols highlight the appearance of the secondary BiNbTe_2_O_8_ orthorhombic phase, after only 20 min of heating treatment. (**b**) Average Raman spectra recorded for both the cubic (bottom) and orthorhombic (top) phases. Insert: Sketch describing the cross-section of a bulk laser ceramic sample and indicating the location of the probed areas for the Raman analysis, spotted by the (x) and (•) symbols.

Figure 5(a) represents the XRD data collected at 510 °C, under atmospheric conditions, as a function of the dwelling time and starting from powder glass. After 0 min spent at this temperature, the initial powder glass has already transformed into the cubic Bi_0.8_Nb_0.8_Te_2.4_O_8_ crystal phase (Ia-3 space group, *a* = 11.2 Å), thus confirming the congruent crystallization process (see^26^). However, after only 20 min spent at 510 °C, the cubic phase starts to partially decompose into the BiNbTe_2_O_8_ orthorhombic phase. In fact, this decomposition is very limited and occurs only at the surface, with a depth that remains to be quantified accurately. It is also important to point out that XRD measurements conducted on a bulk sample lead to the exact same observations. Obviously, in the case of a bulk sample, one can easily get rid of such secondary orthorhombic phase by a simple polishing step (action that was actually done prior to measure the optical transmission of the ceramic samples). Finally, these experiments also reflect the necessity for a careful control of the ceramization process (short time in particular) in order to avoid, or at least minimize, the presence at the surface of this orthorhombic phase.

Another important aspect to consider is the multi-scale organization of such ceramic materials. Indeed, though the grains appear to be ~3–5 μm in size (according to Fig. 1(b)), they do not correspond to single crystals but are polycrystalline and constituted of much smaller crystallites. In fact, using the well-known Scherrer formula^29^, an approximate size of ~36 nm for the coherent domains was extracted from the evaluation of the integral breadth of XRD Bragg peaks (using the powder XRD data collected at 510 °C, for a “dwelling time of 0 min”). Direct observations by Transmission Electron Microscopy (data not shown here, but to be discussed in a forthcoming paper) fully confirm such indirect deduction.

Raman experiments were also conducted on the ceramic sample synthesized after 5 min at 510 °C. One sample has been sliced into two pieces, along the height, and polished. Five different “macroscopic areas” (spotted in the insert of Fig. 5(b), using a (x) symbol) were then selected for a spatial analysis of the structure of the whole sample, especially at its core. For every “macroscopic area”, five acquisitions in five slightly different spatial positions were runned. For the totality of the twenty-five collected Raman spectra, only some minor variations were detected, and one average Raman spectrum is thus presented in this paper (see bottom spectrum in Fig. 5(b), typical of the cubic I*a-3* phase). Hence, whatever the analyzed (x) area, Raman spectra are identical, testifying that the ceramization process is homogeneous at the micron scale and complete after this short heating treatment, even at the core of the sample. Five others Raman spectra were also collected at different locations at the surface of the sample (spotted in Fig. 5(b), using a (•) symbol) and averaged (see top spectrum in Fig. 5(b), typical of the secondary BiNbTe_2_O_8_ orthorhombic P*bca* phase). Figure 5(b) attests then the strong similarities (at least at short and medium range orders) between the cubic and orthorhombic phases, but at the same time definitely reveals real structural differences. The main point to consider remains finally the difference in the spatial location for these two phases; the orthorhombic phase developing only at the surface, if the heating treatment remains correctly time and temperature controlled.

**Figure 6:**
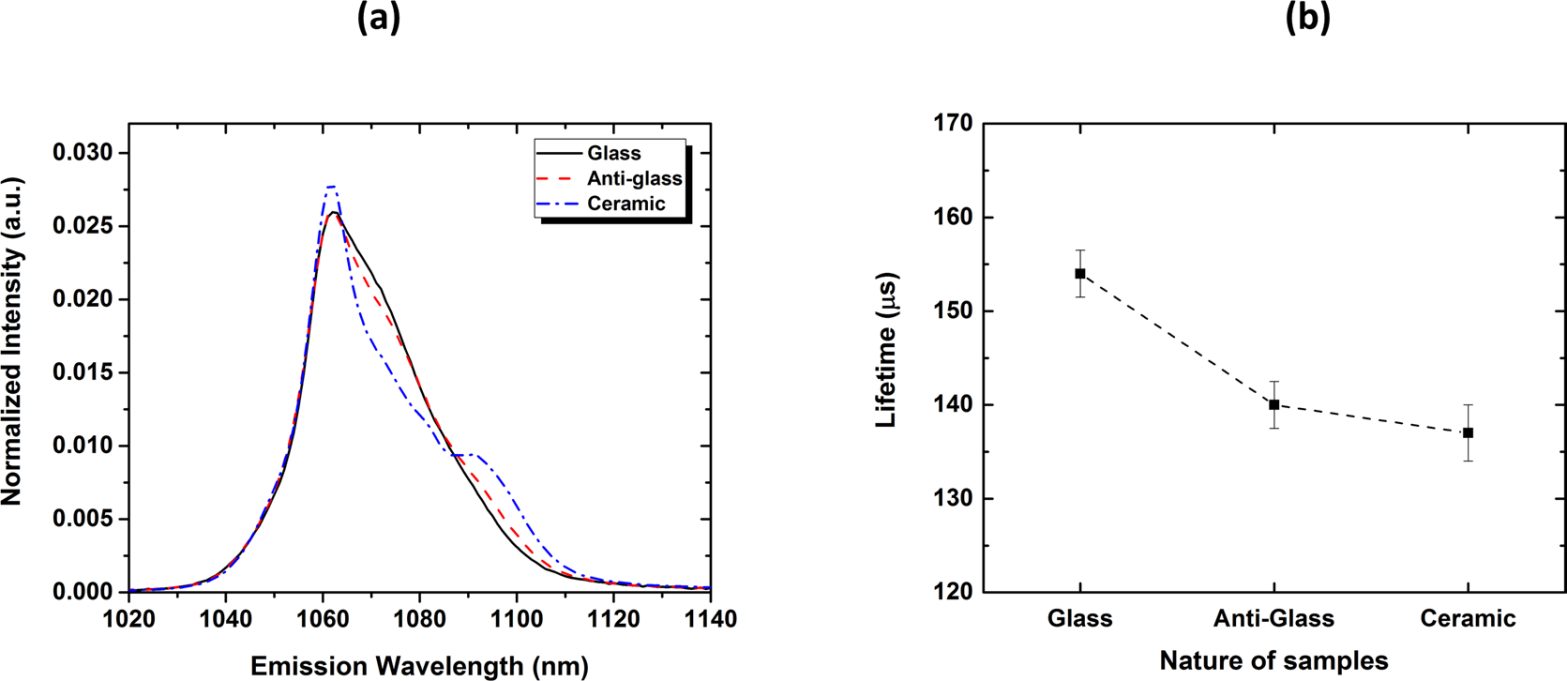
(**a**) Normalized PL emission spectra collected in the range of the ^4^F_3/2_–4I_11/2_ transition, for the glass, anti-glass and ceramic samples, for an excitation wavelength λ_exc_ = 802 nm. (**b**) Evolution of the lifetime value as a function of the nature of the sample (glass, anti-glass or ceramic samples).

Finally, prior to run any lasing tests (see *Part B*), it is mandatory to evaluate the spontaneous light emission properties of these Nd^3+^-doped materials. Hence, in Fig. 6(a) are plotted the PL emission spectra measured at RT for the glass, the anti-glass and the ceramic samples (5 and 15 min samples display the same emission spectra). The anti-glass corresponds actually to the intermediate cubic phase Bi_0.8_Nb_0.8_Te_2.4_O_8_ (space group *Fm*-3*m*, *a* = 5.6043 Å) that was previously evoked in^26^ and which crystallizes at lower temperature than 510 °C. One can then notice the strong resemblances between the anti-glass and the initial parent glass, in total agreement with reported Raman spectroscopy data and with the fact that the local and medium range orders are very similar^30^. A future contribution will soon specifically address the series of phase transformations occurring with increasing temperature, for that peculiar 75TeO_2_-12.5Bi_2_O_3_-12.5Nb_2_O_5_ composition. The transformation of the anti-glass into the final ceramic is then accompanied with the clear observation of crystal field splitting effects and the appearance of sharper features on the corresponding PL emission spectrum, due to the partial ordering of the cations (see future contribution).

Figure 6(b) indicates the evolution of the lifetime values (extracted from the PL decay curves (not shown here), which can be considered as single exponential – the quality of every fit, given by the r^2^ value, is ~0.9995; a perfect fit corresponding to an ideal value of 1), when moving from the initial parent glass to the final ceramic. One can clearly evidence a reduction of the lifetime value, by ~11%, from 154 ± 2 to 137 ± 3 μs. Most of this reduction occurs with the transformation of the glass into the anti-glass, and the concomitant formation of grains and grain boundaries (not shown here).

### Study of the laser emission in polycrystalline tellurite ceramics

**Figure 7:**
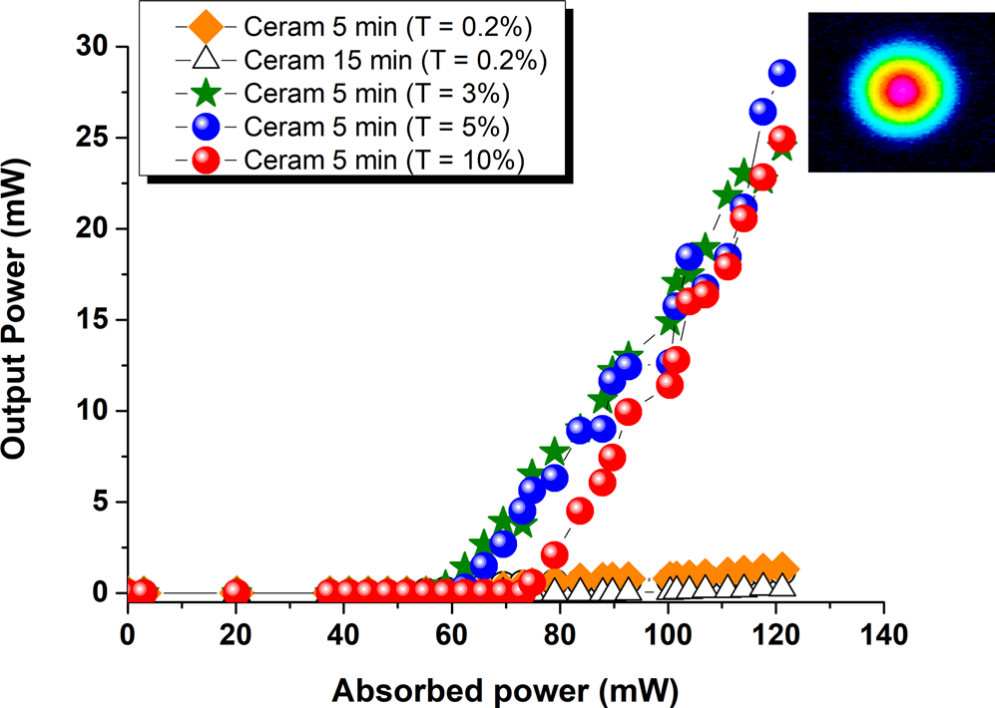
Laser output power at 1064 nm versus the input pump power at 808 nm for different output couplers and for two ceramic samples synthesized after 5 min and 15 min at 510 °C. Inset: image taken by the IR camera of the output beam profile obtained with the short cavity.

In a first step, among the two series of ceramic samples (5 and 15 min heat-treatment at 510 °C), we tried to identify which one exhibited the lower laser power threshold. That experiment was realized using an output coupler transmission of only 0.2% in order to obtain a large number of round trips of the photons in the cavity. In that configuration, laser effect was obtained with 99 mW and 63 mW of absorbed pump power for the ceramic samples synthesized after 15 min and 5 min, respectively (see Fig. 7). Such result falls in complete agreement with the better optical transparency (less scattering) evidenced for the sample which has undergone the shorter heat-treatment under atmospheric conditions (Cf. Fig. 3).

**Figure 8:**
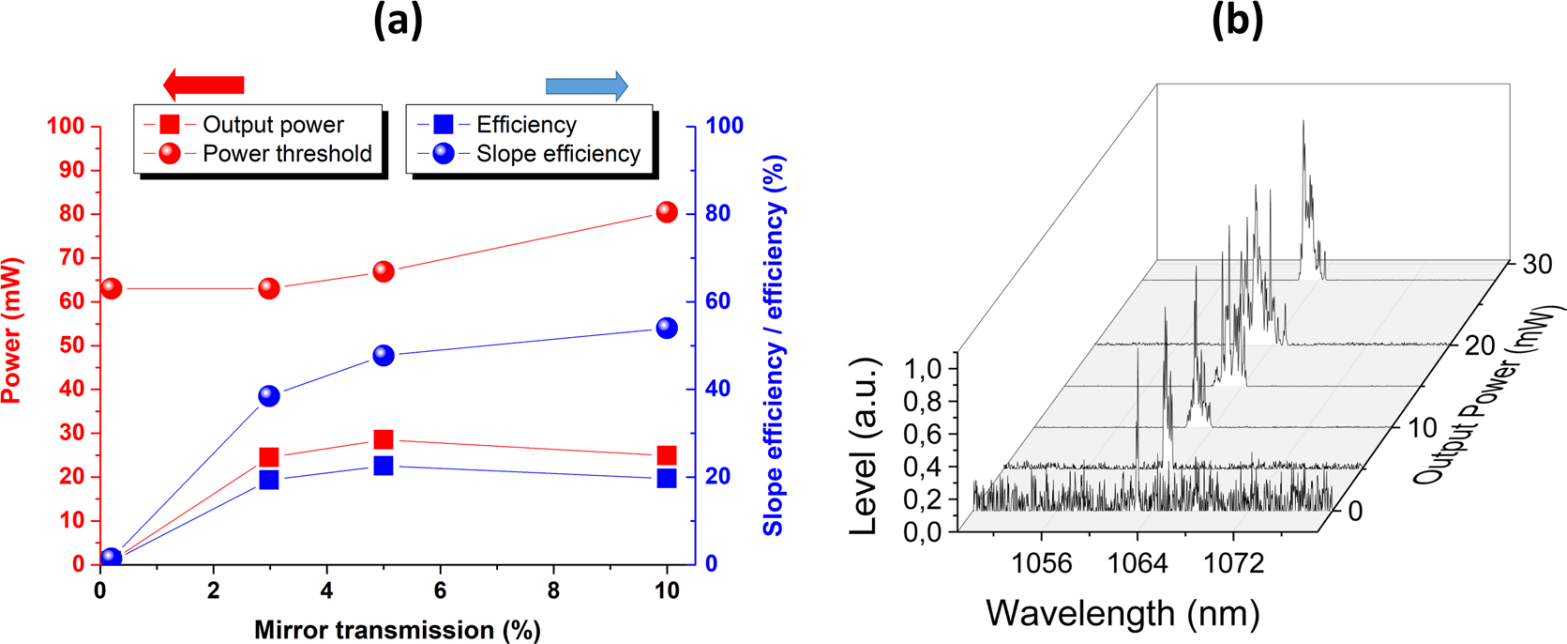
(**a**) Output power, power threshold, efficiency and slope efficiency of the ceramic sample synthesized after 5 min at 510 °C for different output coupler transmissions; (**b**) Spectral evolution of the central laser wavelength versus the output power.

In a second step, the search for the highest laser efficiency was conducted. The output power at 1064 nm was thus measured as a function of the output coupler transmission, only in the case of the ceramic sample heat-treated for 5 min at 510 °C (see Figs 7 and 8). The best results were reached for a maximum absorbed power of 126 mW. An efficiency of 22.5%, which represents more than 28.5 mW of output power at 1064 nm and where the power threshold is close to 66.8 mW, is demonstrated for an output coupler transmission of 5%. Moreover, the best slope efficiency (54%) was obtained for a higher output coupler transmission of 10% (Cf. Fig. 8(a)). For all the experiments, within the range of pump power, the laser beam always keeps a Gaussian shape in the transversal plane (see the insert of Fig. 7), with a central wavelength close to 1063.25 nm (see Fig. 8(b)).

Our results can thus be positioned in respect to all the works previously quoted in the introduction^6–20^. In particular, some emphasis can be made regarding the best performances reached so far by Fusari *et al*., for a 2 wt% Tm^3+^-doped 75TeO_2_-10ZnO-10Na_2_O-5GeO_2_ (mol. %) bulk glass^16^. They indeed reported a (cw) emission, with a maximum output power of 124 mW and a slope efficiency of 28%, for a range of pump threshold of ~150–225 mW. Hence, our data definitely stand among the best (cw) performances obtained so far for bulk laser tellurite materials. In particular, the slope efficiency determined for our transparent ceramics even seems to correspond to the record evaluated for such type of small bulk materials.

Finally, it is important to underline that there is still some room for improvement, as the current performances will be limited by the not perfect transparency (Cf. small amount of light scattering remaining at the emission wavelength of 1064 nm, as evidenced on Fig. 3).

**Figure 9:**
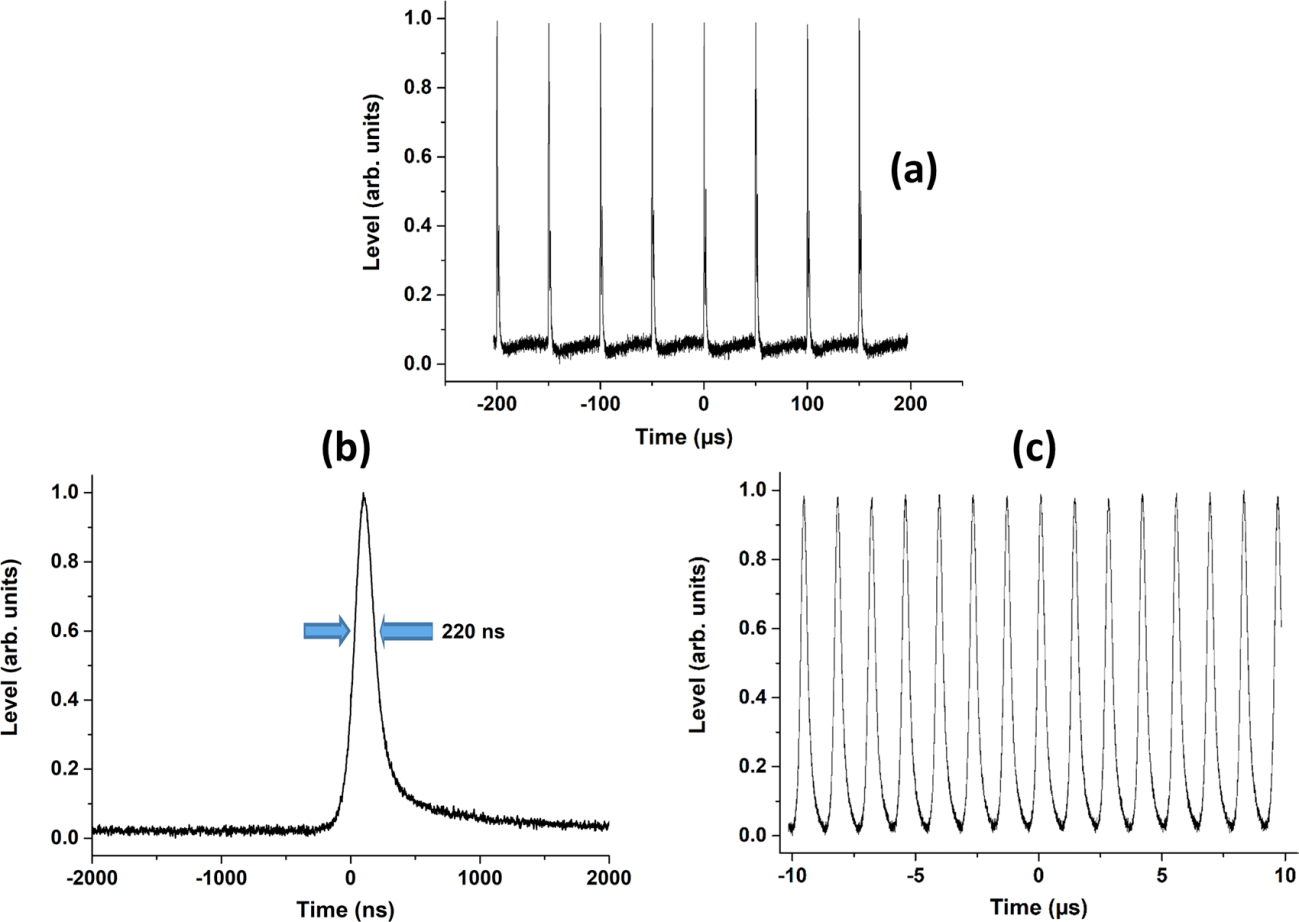
(**a**) Pulsed regime at 20 kHz of repetition rate and for 10% of the output coupler transmission; (**b**) Example of a pulse profile obtained when modulating at 20 kHz; (**c**) Pulse train for a modulation at 728 kHz (pulse duration: 315 ns).

When starting the AOM (Cf. Fig. 2), we also succeeded to run the laser source in gain switched regime between tens of Hz and 728 kHz (maximal frequency reached). The irradiation time has been set in order to obtain a single pulse for each pump period. The shortest output pulse duration of ~220 ns was then obtained for a repetition rate of 20 kHz (see Fig. 9(a,b)). Pulses with duration of ~315 ns were obtained for the highest repetition rate of 728 kHz (see Fig. 9(c)). Under these conditions, the output peak power reached 6.5 W without significant impact of the thermal lens and with a perfect Gaussian beam profile.

## Conclusion

1 mol. % Nd^3+^-doped 75TeO_2_-12.5Bi_2_O_3_-12.5Nb_2_O_5_ glasses were successfully transformed into transparent ceramics, from a fully congruent crystallization process carried out at 510 °C under atmospheric conditions. The necessity of carefully controlling the duration of the heating treatment was proved, in order to avoid the decomposition at the surface of the cubic Bi_0.8_Nb_0.8_Te_2.4_O_8_ *I*a-3 crystal phase into the BiNbTe_2_O_8_ orthorhombic phase. Laser emission was observed in (cw) and gain-switching regimes. A maximum output power of ~28.5 mW, for a laser threshold of ~67 mW, and for an efficiency and slope efficiency of ~22.5 or ~50%, respectively, were obtained in (cw) regime, whereas more than 6.5 W of peak power at a repetition rate of 728 kHz was demonstrated in pulsed conditions.

This work really demonstrates the potential of such polycrystalline transparent ceramics as optically active materials, and position our experimental data as the best results obtained for bulk laser tellurite materials in terms of laser emission slope efficiency. Further improvements of the laser emission performances will now be correlated to an even better control of the fabricated materials. In this regard, a reduction of the dwelling time down to 0 min spent at 510 °C could represent a plausible optimum for the final optical transparency, and simultaneously allow maintaining the full ceramization of the samples.
